# Imaging contrast agent concentration and extracellular volume fraction in the right ventricle

**DOI:** 10.1186/1532-429X-14-S1-O109

**Published:** 2012-02-01

**Authors:** Joseph J Pagano, Kelvin Chow, Ian Paterson, Richard B Thompson

**Affiliations:** 1 Biomedical Engineering, University of Alberta, Edmonton, AB, Canada; 2 Medicine, University of Alberta, Edmonton, AB, Canada

## Background

Globally increased myocardial extracellular volume fraction (ECVF) has been associated with diffuse myocardial fibrosis. ECVF can be estimated using blood and tissue concentrations of gadolinium contrast agent, [Gd], which are calculated using baseline and post-contrast T_1_ values [1]. To date, T_1_ quantification has been limited to the left ventricle (LV) with moderate spatial resolution (~2 mm) and long imaging windows (>200 ms) to accommodate breath-hold acquisitions. These methods have insufficient spatial resolution to image the relatively thin-walled right ventricle (RV). A new cine-imaging approach for the measurement of contrast agent concentration and ECVF using saturation-recovery preparation is evaluated in the LV and RV.

## Methods

A saturation-recovery gated-segmented cine SSFP sequence, similar to the multi-contrast late enhancement imaging method [2], provides a short acquisition window (< 50 ms) enabling end-systolic imaging and higher spatial resolution (~1 mm). Bloch equation simulations of the sequence were used to generate a look-up table to relate the measured ratio of post- to pre-contrast image intensity to the tissue concentration of contrast agent (CLAIR - Contrast Level Assessment using Intensity Ratios). Short axis images were acquired in 9 subjects from an ongoing study of heart failure (Alberta HEART), with contrast-enhanced images at 15 min post 0.15mmol/kg Gadovist. Typical CLAIR pulse sequence parameters: FOV=300mm, 256 matrix, 8 mm slice, flip angle=73°, TE=1.66ms, TR=3.32ms, VPS=14, TI=300ms. Average LV [Gd] in subjects was compared to values obtained using a saturation-recovery SSFP T_1_-mapping sequence [3] calculated using [Gd] = ΔR1/r (ΔR1 = change in 1/T_1_ with contrast, r = relaxivity). For both methods ECVF = (1-Hct)*[Gd]Tissue/[Gd]Blood, with [Gd]Blood obtained via the T_1_ mapping sequence and an assumed Hct of 0.4. Data are presented as mean±SD and differences compared with the two-tailed paired Student’s t-test.

## Results

**Figure 1:**
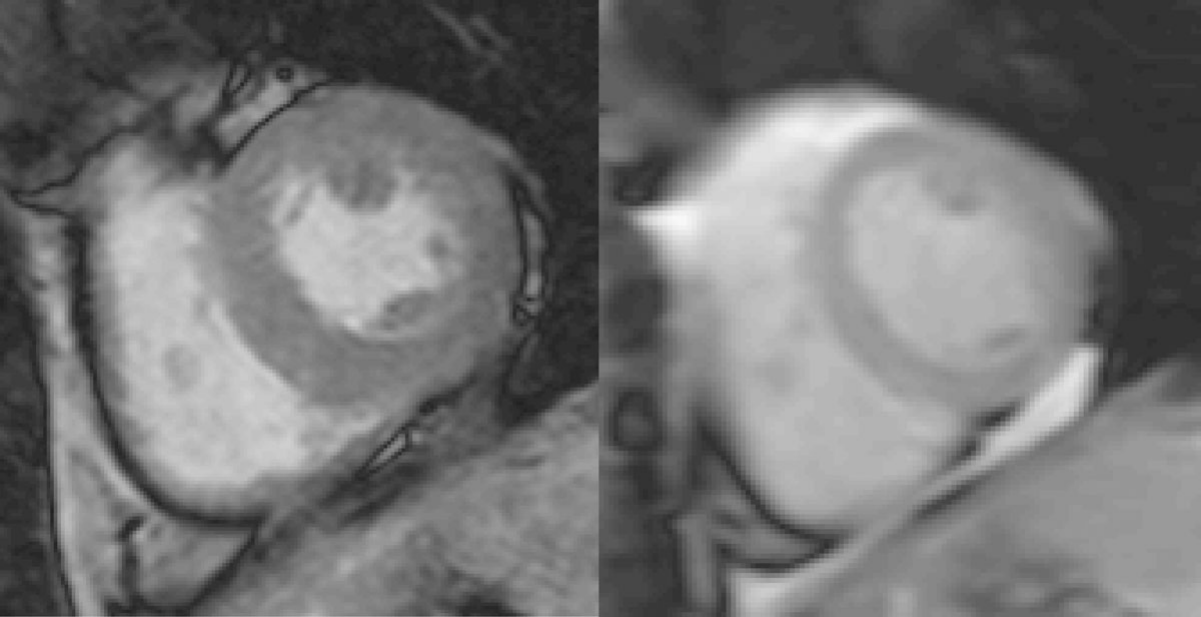
CLAIR image (left) at end-systole (1.17 mm resolution, 46 ms temporal resolution) and conventional T_1_-mapping image at end-diastole (right) (1.88 mm resolution, 225 ms temporal resolution) from the same subject (15 minutes post contrast).

**Figure 2:**
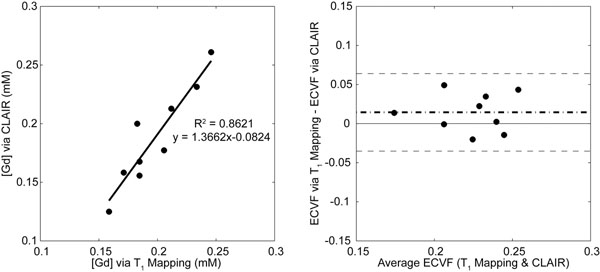
Comparison of CLAIR and conventional T_1_-mapping methods: LV contrast concentration (left) and Bland-Altman plot of extracellular volume fraction (right).

Subject age was 60.7±14.3yrs, with 5 males. Images from an individual using CLAIR (end-systole) and conventional T_1_-mapping (end-diastole) are shown in Fig. 1. LV [Gd] is not statistically different between CLAIR and T_1_ mapping (0.188±0.042 vs. 0.198±0.029 mM, p=0.151) and is significantly correlated between methods (p<0.01) (Fig. 2 left). LV ECVF is not statistically different between CLAIR and T_1_ mapping (0.216±0.027 vs. 0.231±0.027, p=0.120) with negligible bias (Fig. 2 right) between the two methods. CLAIR RV [Gd] (0.212±0.066 mM) and ECVF (0.245±0.054) were not statistically different from LV values (p=0.134 and p=0.093).

## Conclusions

CLAIR yields similar LV myocardial contrast concentration and ECVF in the LV to T_1_-mapping and provides sufficient temporal and spatial resolution for end-systolic RV imaging.

## Funding

CIHR.

Alberta HEART Team Grant.
